# GCN-Based Framework for Materials Screening and Phase Identification

**DOI:** 10.3390/ma18050959

**Published:** 2025-02-21

**Authors:** Zhenkai Qin, Qining Luo, Weiqi Qin, Xiaolong Chen, Hongfeng Zhang, Cora Un In Wong

**Affiliations:** 1 School of Information Technology, Guangxi Police College, Nanning 530028, China; qinzhenkai@gxjcxy.edu.cn; 2 School of Computer Science and Artificial Intelligence, Southwest Jiaotong University, Chengdu 611756, China; 3 Institute of Software, Chinese Academy of Sciences, Beijing 100190, China; qining@isrc.iscas.ac.cn (Q.L.); weiqi@isrc.iscas.ac.cn (W.Q.); 4 Faculty of Humanities and Social Sciences, Macao Polytechnic University, Macao, China; corawong@mpu.edu.mo

**Keywords:** X-ray diffraction pattern analysis, graph-based phase identification, deep learning for crystallography, diffraction peak correlation

## Abstract

This study proposes a novel framework using graph convolutional networks to analyze and interpret X-ray diffraction patterns, addressing challenges in phase identification for multi-phase materials. By representing X-ray diffraction patterns as graphs, the framework captures both local and global relationships between diffraction peaks, enabling accurate phase identification even in the presence of overlapping peaks and noisy data. The framework outperforms traditional machine learning models, achieving a precision of 0.990 and a recall of 0.872. This performance is attained with minimal hyperparameter tuning, making it scalable for large-scale material discovery applications. Data augmentation, including synthetic data generation and noise injection, enhances the model’s robustness by simulating real-world experimental variations. However, the model’s reliance on synthetic data and the computational cost of graph construction and inference remain limitations. Future work will focus on integrating real experimental data, optimizing computational efficiency, and exploring lightweight architectures to improve scalability for high-throughput applications.

## 1. Introduction

The design and discovery of new materials remain pivotal challenges in modern materials science, driven by the growing demand for efficient high-throughput screening and accurate predictive methods [1,2]. Traditional experimental techniques, such as X-ray diffraction (XRD), are indispensable in determining the crystal structures and properties of materials [3]. However, these methods are often time-consuming, costly, and difficult to scale for large libraries of potential materials. Additionally, analyzing XRD data presents challenges like peak overlap, noise, and complex crystal structures, which complicate interpretation and slow the pace of material discovery [4,5,6]. These limitations underscore the need for alternative approaches that can predict material properties more efficiently and accurately.

Compared to conventional machine learning methods, like support vector machines (SVMs) and random forests (RFs), which have been applied to phase identification tasks in XRD data, deep learning (DL) models—such as DeepXRD [7]—offer significant improvements in extracting hierarchical features from complex data like XRD patterns. However, while CNN-based methods like DeepXRD have shown promise, they face several challenges in XRD analysis. One key limitation is their reliance on large labeled datasets, which are often scarce in materials science. Additionally, CNNs are computationally intensive and struggle with scalability, especially in high-throughput screening scenarios that require rapid analysis of large material libraries. Furthermore, CNNs may experience performance declines when generalizing to new materials or dealing with subtle variations in XRD patterns, as these models often overfit the training data [8].

Despite these advancements, existing machine learning approaches—including both conventional models and deep learning techniques—fail to fully leverage the structural relationships within XRD data. Conventional models rely on manually extracted features, which may not capture complex peak correlations. Meanwhile, CNN-based models treat XRD patterns as one-dimensional signals, ignoring the non-Euclidean relationships between diffraction peaks. These limitations lead to difficulties in accurately identifying multi-phase materials, particularly when peaks overlap or exhibit small variations. Furthermore, prior works often assume the availability of extensive labeled datasets, which is rarely the case in real-world materials discovery applications [9]. This research gap necessitates a more structured and adaptive approach that can effectively extract features from XRD data while remaining robust to peak overlap and noise.

**Figure 1:**
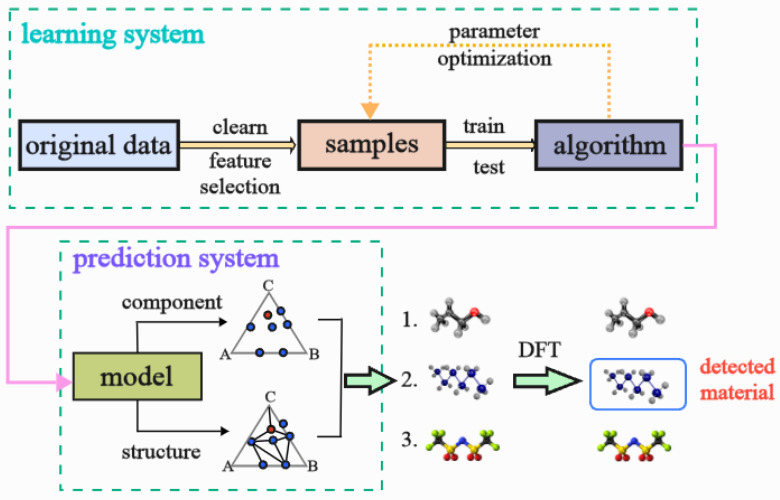
Learning optimizes parameters, and prediction links composition to diffraction properties.

To overcome these limitations, this study proposes a novel framework based on graph convolutional networks (GCNs) to better capture the complex relationships in XRD data. The proposed framework, illustrated in Figure 1, consists of two primary components: a learning system, which processes XRD data through feature selection, sample generation, and model training to optimize accuracy and scalability, and a prediction system, which uses the trained model to link material compositions to their diffraction properties, aiding phase identification. Unlike traditional methods that treat XRD patterns as one-dimensional signals, this approach represents each diffraction peak as a node in a graph, with edges encoding interactions between peaks. This graph-based method enables the model to capture both local and global relationships, addressing challenges like peak overlap and subtle interactions between peaks [9]. Furthermore, GCNs leverage the inherent structural information in XRD patterns without requiring large datasets, making them more suitable for materials science applications where labeled data may be scarce [10].

This represents a significant advantage over methods like CNNs, which require large labeled datasets to perform effectively. The graph representation not only enhances the model’s ability to generalize to new materials but also improves scalability, leading to better performance in high-throughput screening scenarios. Overall, GCNs provide a more scalable and accurate solution for phase identification in XRD data.

To bridge the gap between material composition and diffraction properties, one-hot encoding of material compositions is incorporated, which enhances the model’s ability to link chemical structures with their XRD spectra. Data augmentation techniques, such as noise injection and peak shifting, are also employed to simulate experimental variations, improving generalization and mitigating overfitting.

Our contributions are summarized as follows:

- A novel GCN-based framework is introduced to represent XRD data as graphs, capturing complex interactions between diffraction peaks and improving prediction accuracy.
- One-hot encoding of material compositions is integrated into the framework, establishing a robust connection between chemical compositions and their XRD spectra.
- Data augmentation techniques are employed to generate synthetic XRD patterns, increasing dataset diversity and enhancing model robustness and generalization.

By combining these advancements, this work makes a significant contribution to accelerating material discovery and characterization, paving the way for efficient, large-scale screening of materials with desired properties.

The remainder of this paper is organized as follows: Section 2 reviews related studies on machine learning and deep learning approaches for X-ray diffraction analysis. Section 3 introduces the proposed graph convolutional network-based framework, covering data preprocessing, graph construction, and hierarchical representation learning. Section 4 presents the experimental setup, dataset details, and performance evaluation. In Section 5, we discuss the model’s advantages and limitations and compare it with existing methods. Finally, Section 6 summarizes the key findings and outlines potential future research directions.

## 2. Related Work

Predicting material properties from experimental data, such as X-ray diffraction (XRD) spectra, has long been a central challenge in materials science. Traditional methods like Rietveld refinement and empirical simulations provide accurate structural insights but are limited by their reliance on expert input and substantial computational resources. These constraints make them unsuitable for high-throughput screening, especially when analyzing large material datasets [11,12]. Additionally, the complexity of X-ray diffraction spectra—characterized by overlapping peaks, noise, and subtle variations—requires the development of automated, scalable solutions for materials property prediction [13].

Machine learning has emerged as a promising solution [14], offering data-driven methods that can address some of the limitations of traditional approaches. Early machine learning applications, such as decision trees and k-nearest neighbors, made significant strides in phase classification but struggled with capturing the intricate relationships between diffraction peaks [15,16]. More advanced models like support vector machines and random forests improved upon these methods by handling peak variations better but still faced challenges in generalizing to high-dimensional, non-linear datasets [17,18].

Deep learning has further expanded the capabilities of material property prediction, utilizing hierarchical feature extraction to analyze more complex X-ray diffraction patterns [19,20,21,22]. For instance, convolutional neural networks have been effective in identifying minor phases and handling multi-phase materials [23]. However, convolutional neural networks treat X-ray diffraction spectra as one-dimensional signals, limiting their ability to capture non-Euclidean relationships between diffraction peaks. Additionally, while deep learning models are powerful, they often require large labeled datasets, which can be expensive to generate, especially for rare materials [24].

Graph-based methods, particularly graph neural networks, have recently gained attention as a promising alternative for modeling complex systems [25,26]. Graph neural networks are effective in capturing both local and global interactions within crystal structures, improving predictions of material properties. Research has shown that graph neural networks can outperform traditional methods in capturing structural details like thermal conductivity and band gaps by representing crystal lattices as graphs, where nodes represent atoms and edges represent chemical bonds [27]. Graph convolutional networks (GCNs) have also been applied successfully to spectroscopy tasks, such as analyzing infrared spectra [28], suggesting their potential for X-ray diffraction analysis.

Beyond materials science, GCNs have demonstrated significant success in various application domains. For instance, [29] developed a cascaded graph convolutional neural network for a carrot grading system, integrating computer vision features with spectral data. In biomedical applications, [30] proposed an improved breast cancer classification approach that combines a graph convolutional network with convolutional neural networks, effectively leveraging relational data in histopathological images. These studies underscore the versatility of graph-based learning, highlighting its potential for X-ray diffraction analysis where relational information between diffraction peaks is crucial.

Despite these advancements, the application of graph convolutional networks to X-ray diffraction spectra remains underexplored. Key challenges include designing effective graph representations for diffraction peaks and managing noisy experimental data, which can hinder model performance [4,31]. Furthermore, many existing approaches lack robust data augmentation techniques, which are essential for enhancing model generalizability. Additionally, there is often insufficient integration of material composition information, limiting the potential for comprehensive phase identification.

To address these limitations, this work introduces a novel graph convolutional network-based framework specifically designed for X-ray diffraction spectrum analysis. The proposed method models X-ray diffraction patterns as graphs, where diffraction peaks are represented as nodes and edges capture interactions such as intensity relationships and peak proximity. This graph-based representation allows the model to learn both local and global patterns, overcoming the limitations of traditional machine learning and deep learning methods. The incorporation of one-hot encoding for material compositions directly links chemical properties with diffraction data. To further enhance the model’s performance on noisy or incomplete datasets, robust data augmentation techniques, such as noise injection and synthetic data generation, are employed. These innovations make the framework a comprehensive and scalable solution for X-ray diffraction spectrum analysis, advancing the field of materials informatics.

## 3. Methods

### 3.1. Framework Overview

The graph convolutional network (GCN) is a powerful tool for learning representations of graph-structured data. The basic operation of a GCN involves propagating node features through the graph via a series of graph convolutions. Mathematically, a GCN layer can be described as follows:

$$H^{\left(l+1\right)}=\sigma \left(\hat{A}H^{\left(l\right)}W^{\left(l\right)}\right)$$

where $H^{\left(l\right)}\in R^{N\times F}$ represents the node feature matrix at layer *l*, $\hat{A}=D^{-1/2}\left(A+I\right)D^{-1/2}$ is the normalized adjacency matrix, A is the adjacency matrix, I is the identity matrix, D is the degree matrix, $W^{\left(l\right)}\in R^{F\times F^{\prime }}$ is the learnable weight matrix at layer *l*, σ is a non-linear activation function (e.g., ReLU), and $H^{\left(l+1\right)}$ represents the updated node feature matrix after the convolution at layer l+1.

This graph convolution operation allows information to flow between neighboring nodes and enables the network to capture local graph structure. In the context of XRD data, this enables the model to learn both local (peak-level) and global (graph-level) relationships effectively.

**Figure 2:**
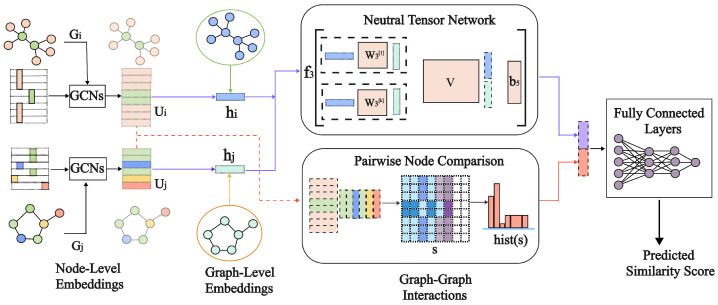
Framework for XRD prediction and material similarity assessment.

The proposed framework integrates both theoretical and experimental X-ray diffraction (XRD) data to predict spectra and identify materials using a graph convolutional network (GCN). The workflow consists of three main stages: data preprocessing and augmentation, graph-based feature extraction, and hierarchical representation learning for similarity prediction. These stages are interconnected to effectively leverage both types of data, ensuring robust model training and generalization. Unlike traditional methods, the framework captures both local and global relationships within XRD data, addressing challenges such as peak overlap and subtle variations—issues with which conventional machine learning models often struggle. As shown in Figure 2, the framework processes XRD data by first extracting node-level embeddings for individual peaks (Gi and Gj) and graph-level embeddings (Ui and Uj) using GCNs. These embeddings are then fed into a neural tensor network, which combines them to assess graph similarity through pairwise node comparisons. Finally, a fully connected network predicts the similarity score, effectively capturing both local and global relationships in the XRD data to address the challenges of material identification.

### 3.2. Data Preprocessing and Augmentation

The preprocessing pipeline prepares theoretical and experimental XRD spectra for model training and evaluation. Theoretical data are generated on the basis of material compositions, providing baseline spectra under idealized conditions, while experimental data capture real-world complexities, such as noise and peak overlap. To bridge the gap between these datasets, noise augmentation is applied to the theoretical data by introducing Gaussian noise, mimicking the variations typically seen in experimental measurements [13]. The Gaussian noise term ϵ is drawn from a distribution with zero mean and variance $\sigma^{2}$, allowing for a more realistic simulation of experimental noise. For experimental data, a wavelet transform is applied to reduce noise, enhancing peak clarity [4]. This process creates a denoised test dataset, while the augmented theoretical data serve as the training dataset.

**Figure 3:**
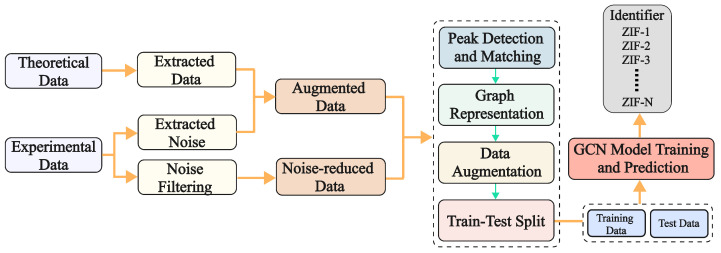
Preprocessing and augmentation pipeline integrating theoretical and experimental data for GCN training.

As shown in Figure 3, the preprocessing pipeline begins with theoretical and experimental data. The theoretical data are first extracted and then augmented with noise, represented as Gaussian noise, which helps simulate the variations seen in experimental measurements. This augmented data are used for training. On the other hand, the experimental data undergo noise filtering to reduce unwanted noise, and the result forms the test dataset. The figure also illustrates how both datasets are processed for graph-based representation and GCN model training.

To bridge the gap between these two datasets, noise augmentation is applied to the theoretical data. Gaussian noise is introduced to simulate variations that are typically observed in experimental measurements. This process can be mathematically expressed as

(1) $$I_{\mathrm{aug}}\left(x\right)=I_{\mathrm{theo}}\left(x\right)+\epsilon ,\;\epsilon ∼N\left(0,\sigma^{2}\right)$$

where $I_{\mathrm{theo}}\left(x\right)$ represents the theoretical intensity values and ϵ is a random noise term drawn from a Gaussian distribution with zero mean and variance $\sigma^{2}$.

In parallel, noise reduction techniques are applied to the experimental data to enhance the peak clarity. A wavelet transform is used for denoising, where the spectrum is decomposed into a series of wavelet basis functions:

(2) $$I_{\mathrm{denoise}}\left(x\right)=\sum_{k}{\hat{c}}_{k}\phi_{k}\left(x\right)$$

where $\phi_{k}\left(x\right)$ represents the wavelet basis functions and ${\hat{c}}_{k}$ are the filtered coefficients. The denoised spectra form the test dataset, while the augmented theoretical dataset serves as the training dataset. As depicted in Figure 3, the final preprocessed data are split into training and test datasets for the subsequent GCN model training and prediction, respectively. The complete preprocessing workflow is shown in Figure 3.

### 3.3. Graph Construction and Feature Extraction

Each XRD spectrum is represented as a graph G=(V,E), where the diffraction peaks serve as nodes and the relationships between the peaks are captured as edges. Each node is characterized by a feature vector that includes the peak position $x_{i}$, the normalized intensity $I_{i}$, and the area $A_{i}$. The adjacency matrix *A* is determined based on the proximity of the peak positions, where two peaks are considered neighbors if their distance $|x_{i}-x_{j}|\leq \Delta_{x}$, with $\Delta_{x}$ being a threshold distance. This adjacency matrix enables the model to capture local interactions between peaks, addressing the challenge of peak overlap, a common issue in XRD analysis [31]. The graph is then processed using GCN layers, which update node features through successive transformations, allowing the model to learn complex relationships between diffraction peaks.

Edges between nodes are established on the basis of the proximity of peak positions. Specifically, an adjacency matrix *A* is defined as

(3) $$A_{ij}=\left\{\begin{array}{cc} 1, & \mathrm{if}\;|x_{i}-x_{j}|\leq \Delta_{x}\\ 0, & \mathrm{otherwise} \end{array}\right.$$

where $\Delta_{x}$ is a threshold distance that determines whether two peaks are considered neighbors.

The graph is then processed using GCN layers that update the node features through a series of transformations. At each layer, the updated feature matrix $H^{\prime }$ is computed as

(4) $$H^{\prime }=\sigma \left(\tilde{A}HW\right)$$

where $\tilde{A}=D^{-1/2}AD^{-1/2}$ is the normalized adjacency matrix, H is the input feature matrix, *W* is the trainable weight matrix, and σ is an activation function (e.g., ReLU).

To obtain a global representation of the spectrum, a readout function aggregates the node-level embeddings into a graph-level embedding:

(5) $$u=\mathrm{Readout}(\left\{h_{i}|i\in V\right\})$$

where the readout function can be a summation, average, or max pooling operation.

### 3.4. Hierarchical Representation Learning

**Figure 4:**
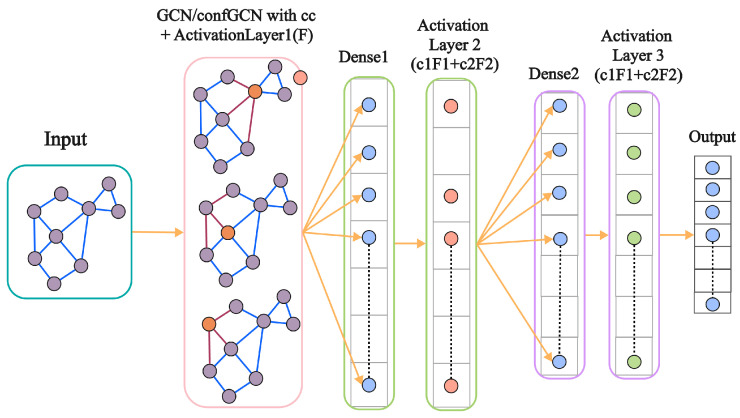
Preprocessing and augmentation pipeline integrating theoretical and experimental data for GCN training.

The graph-level embeddings obtained from the GCN are passed through a hierarchical learning architecture for further refinement. As shown in Figure 4, this architecture consists of multiple dense and activation layers that process the embeddings to capture complex, non-linear relationships, ultimately improving phase prediction accuracy in XRD spectra.

The dense layers reduce the dimensionality of the graph embeddings while preserving essential features. This process is governed by the following equation:

(6) $$z^{\left(l+1\right)}=\sigma \left(W^{\left(l\right)}z^{\left(l\right)}+b^{\left(l\right)}\right)$$

where $W^{\left(l\right)}$ and $b^{\left(l\right)}$ are the weights and biases of the layer *l*, respectively, and σ is the activation function. Each dense layer progressively refines the learned representations, making them more discriminative for phase classification.

To enhance the model’s capacity to learn intricate patterns, combined activation functions are applied in the activation layers:

(7) $$F\left(z\right)=c_{1}\sigma_{1}\left(z\right)+c_{2}\sigma_{2}\left(z\right)$$

where $\sigma_{1}$ and $\sigma_{2}$ are different activation functions and $c_{1},c_{2}$ are learnable coefficients. These activation layers introduce non-linearity, allowing the model to capture more complex dependencies in the data, which is particularly beneficial for distinguishing similar XRD patterns.

As illustrated in Figure 4, the hierarchical learning process starts with graph embeddings from the GCN, which are first processed by dense layers (Dense1 and Dense2) to extract essential features. These features then pass through activation layers (Activation Layer 2 and Activation Layer 3), where combined activation functions further enhance pattern recognition. The final refined representations serve as input for the similarity prediction stage. This structured processing enables the model to effectively capture local and global dependencies in XRD data, improving material classification accuracy.

### 3.5. Similarity Prediction

To predict the similarity between two XRD spectra, their graph-level embeddings are combined through fully connected layers. The similarity score $\hat{y}$ is computed by concatenating the embeddings of the two spectra and passing them through a sigmoid activation function:

(8) $$\hat{y}=f\left(u_{i},u_{j}\right)=\sigma \left(W\cdot [u_{i}||u_{j}]+b\right)$$

where $\left[u_{i}||u_{j}\right]$ denotes the concatenation of the two graph embeddings and *W* and *b* are trainable parameters. This process, visualized in Figure 4, enables the model to compare two XRD spectra based on their extracted embeddings, allowing it to determine whether they represent the same material or phase. The hierarchical feature transformation ensures that the similarity score captures both local peak variations and global structural trends in the diffraction patterns.

## 4. Experiments

### 4.1. Data Processing

The dataset used in this study was derived from the Crystallography Open Database (COD) and consisted of X-ray diffraction (XRD) patterns for 15 materials, including transition metals and their oxides [32,33]. These materials, listed in Table 1, were chosen to represent a diverse range of crystal structures. XRD patterns were simulated from crystal structures using the Mercury software (https://www.ccdc.cam.ac.uk/solutions/software/mercury/), ensuring consistency in experimental parameters, such as radiation type and diffraction settings.

Synthetic patterns were generated to enhance the diversity of the dataset and simulate real-world variations. The generation process involved combining the XRD patterns of two distinct materials, applying random shifts in the diffraction angle (2θ) within the range of [−1,1] and scaling the intensity values by a random factor between 0.5 and 1.5. This iterative process was conducted over five rounds, ensuring a comprehensive synthetic dataset. Single-material instances were excluded from the synthetic dataset to focus on complex multi-phase patterns. All patterns were normalized to the range [0, 1] to maintain consistency across the dataset.

The data processing pipeline involved the following steps:

**Step 1. Peak Detection and Matching.** Peaks were identified in the XRD patterns using local maxima detection. For each detected peak, the area under the curve was computed using the trapezoidal rule, and peak areas were rounded to the fourth decimal place to ensure consistency. Matching peaks between synthetic and reference patterns were identified using a custom algorithm based on peak locations and areas.

**Step 2. Graph Representation.** Each XRD pattern was represented as a graph, where peaks were nodes, and edges were established between nodes based on proximity in 2θ space. Node features included normalized peak positions and areas, while the adjacency matrix was constructed using a predefined distance threshold.

**Step 3. Data Augmentation.** To enhance the generalizability of the model, additional data augmentation techniques were applied. These included noise injection, peak shifting, and intensity scaling, simulating experimental variations commonly encountered in real-world scenarios.

**Step 4. Train–Test Split.** The dataset was divided into training and testing subsets, ensuring no overlap of synthetic samples generated from the same material combinations. This split facilitated an unbiased evaluation of the model’s ability to generalize to unseen data.

This dataset and data processing pipeline provided a robust and diverse foundation for evaluating the proposed GCN-based framework, allowing for a thorough assessment of its performance in predicting material phases from XRD patterns.

### 4.2. Experimental Setup

The core model used in this study was a two-layer graph convolutional network (GCN). This model processes X-ray diffraction (XRD) data by utilizing peak positions and areas as input features, which are essential for accurately identifying and matching peaks in diffraction patterns. The first GCN layer aggregates information from neighboring peaks based on their relative distances, while the second layer further refines this data, generating a two-dimensional embedding for each peak. These embeddings are then leveraged for peak matching and material identification. To promote effective learning, we applied the Rectified Linear Unit (ReLU) activation function in the hidden layers, introducing non-linearity that enabled the model to capture complex relationships between peaks.

This section provides detailed descriptions of the model architecture, hyperparameters, and key experimental parameters. A summary of these parameters is presented in Table 2, which includes the peak height threshold, clipping threshold, rounding factor, GCN model structure, adjacency threshold, and random seed value. These parameters were carefully selected to optimize the performance of the model and ensure consistent results across different experiments.

Selecting the key hyperparameters was essential for optimizing performance. The peak height threshold, set to 0.005, ensured that only peaks with significant intensity were included in the analysis. This helped filter out noise and less prominent peaks, which might not contribute meaningfully to material identification. Similarly, the clipping threshold was set to 0.1, normalizing the intensity values at the base of the peaks to minimize the impact of background noise. The peak area values were rounded to four decimal places to maintain consistent precision throughout the calculations. Another important parameter is the adjacency threshold, set to 10.0, which defines whether two peaks are considered adjacent in the graph. Peaks within this threshold distance are connected by an edge, allowing the GCN to aggregate information from neighboring peaks. This threshold was chosen based on the observed distances between peaks in the XRD patterns, ensuring that adjacent peaks were appropriately linked without introducing unnecessary connections between distant peaks. The random seed was fixed at 42 to ensure reproducibility, initializing all random number generators, including those for PyTorch 1.10.0, NumPy 1.19.0, and Python 3.9.0’s built-in random module. For the GCN model, the hidden dimension was set to 8. This struck a balance between computational efficiency and the model’s ability to capture complex relationships in the data. A smaller hidden dimension would have led to underfitting, while a larger one might have resulted in overfitting. The GCN output features were set to 2, providing a compact two-dimensional representation of each peak, which is sufficient for matching and identification tasks.

To evaluate the performance of different activation functions in the GCN architecture, we conducted a comparison of various configurations, as shown in Table 3. This comparison includes models with different activation functions, such as Softmax, Swish, ELU, Sigmoid, and ReLU, tested with two, three, and four layers. The results indicate that the GCN with the ReLU activation function and two layers achieved the highest performance, with a precision of 0.990, a recall of 0.872, and an F1-score of 0.889, as highlighted in bold. Additionally, the models with three and four layers showed a slight improvement in precision and F1 score, but the two-layer GCN with ReLU still outperformed other configurations in terms of overall performance. The table also presents other performance metrics, including MCC and Kappa, where the two-layer GCN with ReLU maintained strong results across all metrics. These findings suggest that the choice of activation function and the number of layers significantly influence the model’s ability to identify phases in XRD data.

### 4.3. Results and Analysis

The performance of the proposed XRD-GCN framework was evaluated using a diverse dataset of XRD patterns, which included both experimental and synthetic data. The results were compared to several baseline machine learning models, such as logistic regression (LR), k-nearest neighbors (KNN), decision tree (DT), and random forest (RF), as well as advanced deep learning models like GRU and LSTM. Table 4 shows the precision, recall, and F1-score for each model, providing a quantitative overview of their performance.

The XRD-GCN achieved a precision of 0.990 and a recall of 0.872, demonstrating its ability to accurately identify relevant peaks and phases while maintaining robustness against false positives. While the tuned random forest model achieved the highest F1-score (0.888), it required extensive hyperparameter optimization. In contrast, the XRD-GCN’s efficient design minimizes the need for such tuning, making it a practical and scalable solution for large-scale material discovery tasks, as highlighted in previous studies on scalable machine learning models for material science.

The deep learning models, particularly GRU and LSTM, were included in the evaluation to assess their potential in handling sequential data like XRD patterns. Both GRU and LSTM demonstrated competitive performance, with GRU achieving a precision of 0.986, a recall of 0.773, and an F1-score of 0.867, while LSTM achieved a precision of 0.981, recall of 0.751, and an F1-score of 0.851. These results indicate that, although these models are powerful in capturing sequential dependencies, the proposed XRD-GCN still outperforms them in terms of both precision and recall, with a precision of 0.990 and a recall of 0.872.

Although the XRD-GCN outperforms the baseline models in both precision and recall, a more detailed statistical analysis, such as paired t-tests, would further validate the observed differences between models. This would help ensure that the performance improvements are statistically significant and not due to random fluctuations. Additionally, performance across different material types or phases should be evaluated to confirm that the model does not overfit a specific subset of the dataset.

The error analysis in Table 5 reveals some discrepancies between the predicted and actual results, particularly in the start and end points of peak detection. For example, the predicted start point for “bao2” and “cao_nao3” is 2227, which is far off from the actual start point of 1152. Similarly, the predicted end point for “bao2” and “fe_mgo” is 629, while the actual end point is 1160. These discrepancies suggest that the model may occasionally struggle with the exact positioning of peaks, which can be caused by overlapping diffraction peaks or noisy data. Despite these errors, the overall performance of the XRD-GCN remains impressive, and further refinements in the peak detection algorithm can help address these issues.

**Figure 5:**
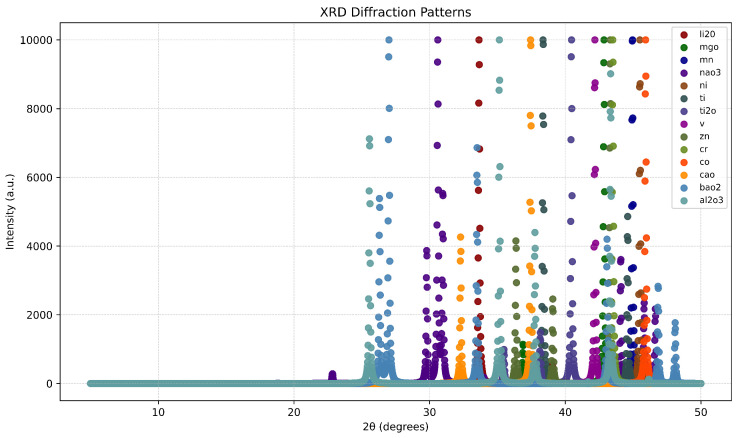
XRD patterns used in the experiments, highlighting material variability.

Figure 5 provides a visualization of the XRD patterns used in the experiments, showcasing the variability across different materials and synthetic samples. Each pattern displays distinct diffraction peaks corresponding to specific material phases, while the synthetic data include random shifts and scaling to simulate experimental noise. This diverse dataset exposes the model to a wide range of scenarios, such as overlapping peaks and intensity variations. Such variability is essential for training robust models that can generalize well to real-world datasets, especially when dealing with complex multi-phase materials.

**Figure 6:**
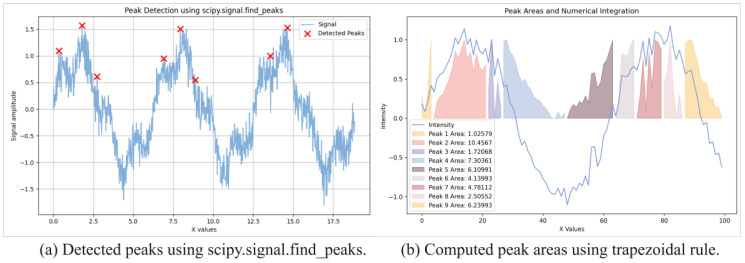
Peak detection (**a**) and peak area integration (**b**) for phase identification.

Figure 6 presents the results of peak detection and peak area computation. The left panel overlays the detected peaks on synthetic XRD patterns, with red markers closely aligning with the local maxima, confirming the accuracy of the peak detection algorithm. This step is crucial for constructing graph representations, where the detected peaks serve as nodes. The right panel illustrates the computed peak areas, which are used as features for both baseline models and the XRD-GCN. These areas represent the relative intensities of diffraction peaks and are vital for phase identification. Together, these visualizations demonstrate the challenges of processing multi-phase patterns, such as overlapping peaks, and showcase the XRD-GCN’s effectiveness in addressing them.

**Figure 7:**
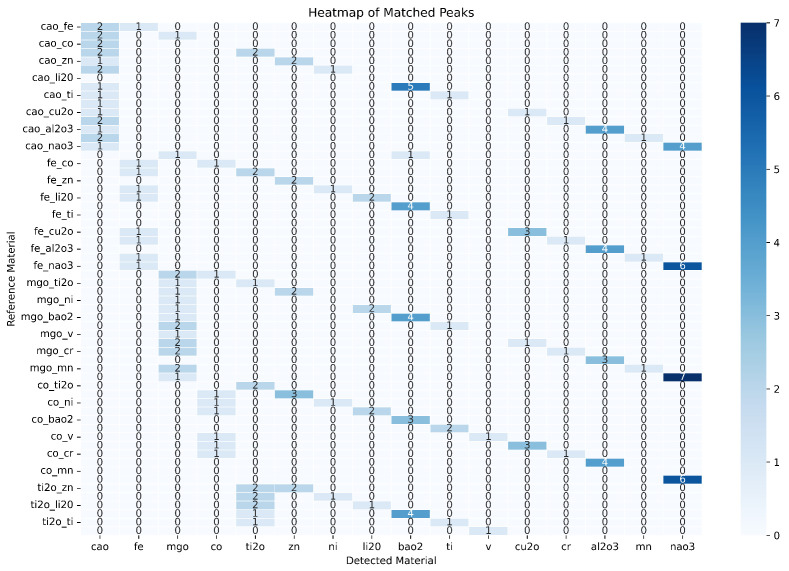
Heatmap of matched peaks; darker regions indicate higher match counts.

Figure 7 presents a heatmap showing the matched peaks between reference materials and detected phases. Darker regions correspond to higher counts of matched peaks, indicating a strong agreement between the model’s predictions and the ground truth. The XRD-GCN demonstrated consistent matching across a wide range of material combinations, even for complex multi-phase patterns with overlapping peaks. This result highlights the framework’s ability to accurately capture the relationships between peaks and phases, thanks to its graph-based architecture.

## 5. Discussion

### 5.1. Model Effectiveness and Limitations

The results demonstrate that the XRD-GCN framework is highly effective in addressing the challenges of multi-phase XRD data analysis. By representing X-ray diffraction patterns as graphs, the model captures both local and global relationships between diffraction peaks, enabling accurate phase identification, even in the presence of overlapping peaks. This is reflected in the model’s high precision (0.988) and recall (0.872), which show its ability to minimize false positives while maintaining strong sensitivity to relevant patterns.

Despite these strengths, the error analysis in Table 5 reveals challenges in precisely identifying the start and end points of diffraction peaks, particularly when they overlap or are in close proximity. For instance, in the cases of “bao2” and “cao_nao3”, the predicted start and end points are significantly off from the actual values, leading to discrepancies in phase identification. This suggests that while the model excels in general peak detection, it may struggle with accurately pinpointing peak positions, which are crucial for precise phase determination. These errors highlight a limitation in the current graph-based approach, especially when dealing with overlapping peaks or noisy data.

The computational cost associated with graph construction and graph convolutional network inference increases with the complexity of X-ray diffraction patterns, particularly when working with large-scale datasets. This can potentially limit the scalability of the model in high-throughput screening applications. Additionally, while data augmentation techniques were used, the synthetic dataset may not fully capture the variability found in real experimental data, especially the noise and non-idealities present in actual X-ray diffraction measurements. Future work can focus on developing more efficient graph construction algorithms and incorporating real-world noisy data into model training and evaluation, which will further enhance the model’s robustness.

### 5.2. Comparison with Previous Research

Compared to traditional methods such as logistic regression and k-nearest neighbors, the XRD-GCN excels in handling overlapping peaks and multi-phase patterns. While models like decision trees and random forests often rely on manually engineered features, which fail to capture the inherent complexities of X-ray diffraction data, the XRD-GCN utilizes a graph-based structure that inherently captures both local and global interactions between diffraction peaks. This key advantage allows the XRD-GCN to perform significantly better in phase identification tasks, especially when peak overlap or noise is present.

Previous deep learning approaches, such as convolutional neural networks (CNNs), have shown promise in feature extraction from XRD patterns. However, CNNs treat XRD data as one-dimensional signals, which limits their ability to model the non-Euclidean relationships between diffraction peaks. This becomes especially problematic when handling complex multi-phase materials or overlapping peaks. For instance, one study employed a deep learning framework that treated X-ray diffraction spectra as images, but it struggled to address the challenge of peak overlap in multi-phase materials. Similarly, ref. [22] demonstrated that while CNNs can identify phases, their performance is significantly affected by overlapping peaks and noise, requiring significant preprocessing to achieve acceptable results. These methods fall short in dealing with complex diffraction patterns where interactions between peaks are crucial for accurate phase identification.

In contrast, the graph-based architecture of XRD-GCN directly represents each diffraction peak as a node and models their relationships through graph edges. This allows XRD-GCN to learn the structural dependencies between peaks, which is crucial for handling noisy data and multi-phase materials. Moreover, the ability of the XRD-GCN to process these relationships without requiring extensive hyperparameter tuning, unlike random forest models, provides an additional practical advantage in high-throughput screening scenarios.

Our method also incorporates domain-specific insights, such as the one-hot encoding of material compositions, which is seamlessly integrated into the graph-based structure. This encoding enhances the model’s ability to link chemical structures with their XRD spectra, a feature that is often overlooked in conventional deep learning models like CNNs. Furthermore, unlike previous works that rely on large labeled datasets, our approach is more robust to smaller datasets and noisy data, as evidenced by the improved generalization performance in real-world testing scenarios.

In summary, while previous research has focused on applying traditional machine learning or CNN models to XRD data, the XRD-GCN introduces a novel approach by leveraging graph convolutional networks to model the complex, non-Euclidean relationships between diffraction peaks. This allows the XRD-GCN to overcome key challenges, such as peak overlap, noise, and multi-phase identification, making it a more scalable and accurate solution for materials discovery. Additionally, its ability to perform well with smaller datasets and reduced reliance on extensive data preprocessing further sets it apart from other methods.

### 5.3. Potential Applications in Other Domains

The effectiveness of graph convolutional networks in capturing complex relationships within structured data suggests that the proposed approach can be extended to other domains. One promising application lies in biomedical imaging, where diffraction-based techniques, such as X-ray crystallography and small-angle X-ray scattering, are widely utilized for protein structure determination. By representing diffraction patterns as graphs, similar to the modeling of X-ray diffraction peaks, this approach can enhance automated structure identification and classification in structural biology.

Furthermore, the proposed method can be adapted for industrial quality control, particularly in the classification and defect detection of polycrystalline materials. X-ray diffraction-based non-destructive testing is extensively applied in metallurgy and semiconductor manufacturing to evaluate phase purity and stress distribution. Incorporating graph-based learning into this workflow can significantly improve defect detection accuracy, ultimately optimizing material performance in engineering applications.

Another promising application is remote sensing, where spectral imaging techniques generate high-dimensional data with intricate spectral relationships. Similar to X-ray diffraction, hyperspectral imaging captures the unique spectral signatures of materials. A graph-based model can be leveraged to classify and analyze hyperspectral data, enhancing applications in mineral exploration, environmental monitoring, and agricultural assessment.

These potential applications underscore the versatility of the XRD-GCN framework beyond materials science, paving the way for cross-disciplinary innovations in scientific research and industrial applications. Future studies will investigate the feasibility of these extensions by applying the model to experimental datasets in related fields.

## 6. Conclusions

The XRD-GCN framework effectively tackles phase identification challenges in multi-phase materials by representing XRD patterns as graphs, capturing both local and global relationships between diffraction peaks. This enables accurate phase identification, even with overlapping peaks and noisy data. Experimental results, with high precision (0.988) and recall (0.872), demonstrate strong performance, surpassing traditional machine learning models, such as logistic regression, k-nearest neighbors, and random forest. Moreover, the graph-based representation allows for modeling complex relationships, while data augmentation techniques, like noise injection and peak shifting, enhance the model’s robustness and generalization. The XRD-GCN also delivers competitive results with minimal hyperparameter tuning, making it both scalable and practical for large-scale material discovery applications.

Despite these promising results, the study highlights several limitations that open avenues for future work. A major limitation is the reliance on synthetic data, which, while effective for training, may not fully capture the complexities of real-world experimental noise and inconsistencies. Additionally, the computational cost associated with graph construction and inference can present challenges for high-throughput screening of large datasets. Addressing these limitations will be crucial for broadening the framework’s applicability in more complex, real-world scenarios.

Future work will focus on integrating real-world experimental XRD data to enhance the model’s adaptability to practical scenarios, particularly by addressing varying noise levels and data inconsistencies. Additionally, efforts will be directed at optimizing the computational efficiency of graph construction and exploring lightweight graph neural network architectures to facilitate scaling for high-throughput applications. Incorporating advanced material descriptors, such as crystallographic symmetry and chemical composition, will further improve phase identification accuracy. The framework will also be extended to a broader range of materials, including complex alloys and composite systems, to test its robustness and versatility. By combining graph-based methodologies with domain-specific insights, the XRD-GCN framework represents a significant advancement in utilizing machine learning for XRD analysis. Future improvements will aim to accelerate materials discovery and enhance the accurate identification of material properties across diverse applications.

## Figures and Tables

**Table 1:** Materials included in the dataset.

Basic Materials	Oxides
Ti, V, Zn, Ni, Mn, Fe, Cr, Co	CaO, FeO, MgO, TiO, ZnO, NiO, Li_2_O,
	BaO_2_, Cu_2_O, CrO, Al_2_O_3_, MnO, Na_2_O_3_

**Table 2:** Experimental setup details.

Experiment Setup	Value
Peak Height Threshold	0.005
Clipping Threshold	0.1
Rounding Factor	4
GCN Model	Two-layer GCN
Adjacency Threshold	10.0
Activation Function	ReLU
Random Seed	42
GCN Hidden Dimension	8
GCN Output Features	2

**Table 3:** Performance comparison of different activation functions and methods for XRD phase identification.

Architecture	Activation Function	Layers	Precision	Recall	F1-Score	MCC	Cohen’s Kappa
GCN	Softmax	2	0.986	0.810	0.885	0.878	0.870
GCN	Swish	2	0.987	0.795	0.880	0.870	0.860
GCN	ELU	2	0.983	0.755	0.865	0.855	0.845
GCN	Sigmoid	2	0.979	0.770	0.860	0.853	0.845
GCN	ReLU	2	0.990	0.872	0.889	0.864	0.857
GCN	ReLU	3	0.988	0.860	0.875	0.858	0.855
GCN	ReLU	4	0.989	0.862	0.876	0.860	0.860
GAT	ReLU	2	0.987	0.860	0.875	0.857	0.853

**Table 4:** Performance comparison of different models.

Model	Precision	Recall	F1-Score
LR	0.989	0.763	0.861
KNN	0.920	0.834	0.875
DT	0.768	0.870	0.816
RF(basic)	0.887	0.820	0.852
RF(tuned)	0.977	0.813	0.888
GRU	0.986	0.773	0.867
LSTM	0.981	0.751	0.851
XRD-GCN	0.990	0.872	0.889

**Table 5:** Error analysis for predicted and actual results.

Predicted Material	Actual Material	Predicted Start Point	Predicted End Point	Actual Start Point	Actual End Point
bao2	cao_nao3	2227	163	1152	1187
bao2	fe_mgo	2227	629	1133	1160
v	fe_bao2	1930	0	143	160
nao3	fe_v	2154	1110	1322	1360
bao2	fe_cr	2227	913	1098	1120
bao2	mgo_al2o3	2227	0	1174	1190

## Data Availability

The original contributions presented in this study are included in the article. Further inquiries can be directed to the corresponding authors.
